# Complex regional pain syndrome and use of psychotropic drugs as a proxy for psychological health

**DOI:** 10.1038/s41598-025-09701-9

**Published:** 2025-07-10

**Authors:** Lars B. Dahlin, Raquel Perez, Erika Nyman, Malin Zimmerman, Juan Merlo

**Affiliations:** 1 Department of Translational Medicine – Hand Surgery, Lund University, Skåne University Hospital, Jan Waldenströms g 5, SE-20502 Malmö, Sweden; 2 Department of Hand Surgery, Skåne University Hospital, SE-20502 Malmö, Sweden; 3 Department of Biomedical and Clinical Sciences, Linköping University, SE- 58183 Linköping, Sweden; 4 Unit for Social Epidemiology, Department of Clinical Sciences (Malmö), Faculty of Medicine, Lund University, SE-20502 Malmö, Sweden; 5 Department of Hand Surgery, Plastic Surgery and Burns, Linköping University Hospital, SE-58183 Linköping, Sweden; 6 Center for Primary Health Research, Region Skåne, Malmö, SE-20502 Sweden; 7 Department of Orthopedics, Helsingborg Hospital, SE-25187 Helsingborg, Sweden

**Keywords:** Chronic pain, Trauma, Neuropathic pain, Peripheral neuropathies

## Abstract

We aimed to investigate the association between psychotropic drug use (proxy for psychological health) and Complex Regional Pain Syndrome (CRPS) type 1 and type 2 in upper and lower limbs. We also assessed the impact of demographic and socioeconomic factors. From a national record linkage database [4,706,821 individuals (25–64 years); CRPS type 1 = 809; type 2 = 225], prevalence ratios (PR), absolute risk (AR), and AR difference (ARD) [95% confidence intervals (CI)] were estimated by logistic and Cox regressions with constant time at risk. About 36% of CRPS individuals consumed psychotropic drugs (reference: general population 15%). Unadjusted PRs were two times higher in nerve injuries/disorders without CRPS [PR = 1.78; 95% CI: 1.73–1.82] and CRPS type 1 [2.33 (2.07–2.62)] and almost three times higher in CRPS type 2 [2.79 (2.27–3.42)]. Higher ARDs were observed in type 2, independent of level of income, and were higher for the high-income category. In type 2, ARDs were higher for middle-high/high occupational status levels. Higher ARDs were observed in nerve injuries/nerve disorders, CRPS type 1, and type 2 in immigrants, especially high for type 2. Regarding most age intervals, ARDs were higher for individuals with CRPS type 2. Individuals with CRPS, especially type 2, have high risk of impaired psychological health.

**Supplementary Information:**

The online version contains supplementary material available at 10.1038/s41598-025-09701-9.

## Introduction

Complex Regional Pain Syndrome (CRPS), a severe and chronic primary pain disorder, with recently updated subtypes, can be induced by various traumas. The diagnosis is based on internationally agreed Budapest criteria and the classification will be updated in International Classification of Diseases, 11th Revision (ICD-11)^1–4^. CRPS is defined as pain disproportionate to the possible previous events or trauma, including injury to at least one peripheral nerve, which may also include nerve disorders, such as nerve entrapment disorders (CRPS type 2), or with no evident nerve injury (CRPS type 1). The distinction is uncertain where it is reported that the symptoms and diagnostic signs of type 1 and type 2 are considered identical^4^. Furthermore, the feature of CRPS type 2 is that symptoms and signs extend beyond the area of the injured nerve, thereby with a distinction from neuropathic pain^4–6^. Other differential diagnoses should be excluded before a CRPS diagnosis. The condition may affect the upper and lower limbs with a reported distribution of up to 4:1^1,7^. Risk factors for developing the condition, including blunt traumatic extremity injuries, fractures (particularly distal radius fractures; potentially related to high risk for CRPS in women), sprains, different surgical procedures, nerve injuries like carpal tunnel syndrome (CTS), fibromyalgia, migraine, cigarette smoking, ACE inhibitor therapy and less frequently seen in rheumatoid arthritis, have been vaguely identified^1,2,4,8–11^.

It is plausible that CRPS affects an individual´s mental health and quality of life, which in turn may impact the recovery from CRPS. However, any association between psychological health and CRPS is complex since there is a described bidirectional relationship between pain and mental illness, showing an increased incidence rate ratio for developing mental illness after pain and vice versa^12^. Thus, one may argue that the status of the individual´s psychological health (a) may have an impact on (risk for) the development of CRPS and/or (b) that the psychological health may be severely impaired after the individual is affected by CRPS, but the exact mechanism(s) behind this relationship is not clarified. As reported in some literature, “anxiety and depression” may be linked to CRPS^1,13–20^ without any proof of being causative. Nevertheless, data suggest that “psychological behavior, depression, and preoperative psychological distress or an increased pain level” are not predictive for the development of CRPS^2,10,21^; thus, not being causative since causation requires specific criteria^22^.

Consumption of psychotropic drugs (e.g., psycholeptics, antidepressants, or psycholeptics and psychoanaleptics in combination) is a valid proxy for impaired psychological health^23,24^. Amitriptyline, together with pregabalin and gabapentin, are drugs that can be used for treatment of CRPS. Amitriptyline, duloxetine, and venlafaxine are drugs that one here may interpret as psychoactive rather than psychotropic^4,16^. An increased use of psychotropic drugs in individuals with CRPS, or in individuals with nerve injuries, which may also include injury from a nerve entrapment disorder, in the upper or lower limbs may be expected. However, this association has not previously been investigated. In addition, such associations could be different in diverse socioeconomic groups. Therefore, we aimed to compare the use of psychotropic drugs in relation to the presence or absence of CRPS, with and without associated nerve injury (type 2 and 1, respectively) and among individuals with a nerve injury, with and without surgical intervention, as well as overall in the population and in strata defined by demographical and socioeconomic factors.

## Methods

### Population & methods

#### Databases

The present study joins data as previously described^23–25^ from several registers with information at the individual level, which covers the entire population of Sweden. Data were used from the registers of the Total Swedish Population (TPR) and the Longitudinal Integration Database for Health Insurance and Labour Market Studies (LISA), administered by Statistics Sweden (www.scb.se/en/) and contain information on demographic and socioeconomic factors. This information was linked to registers administered by the National Board of Health and Welfare (www.socialstyrelsen.se/en/), including the National Patient Register (NPR), recording information on discharge diagnoses and surgical procedures, as well as the Cause of Death Register (CDR) and the Swedish Prescribed Drug Register (SPDR). The last contains information about all drug dispensations in Swedish pharmacies, except stockpiles in nursing homes and hospital wards.

The authorities anonymized the registers after initial approval by the Regional Ethical Committee (see below under *Ethics*) and revision and consent were provided by the registers´ data safety committees. The record linkage was performed by us using a unique anonymized personal identification number.

**Fig. 1:**
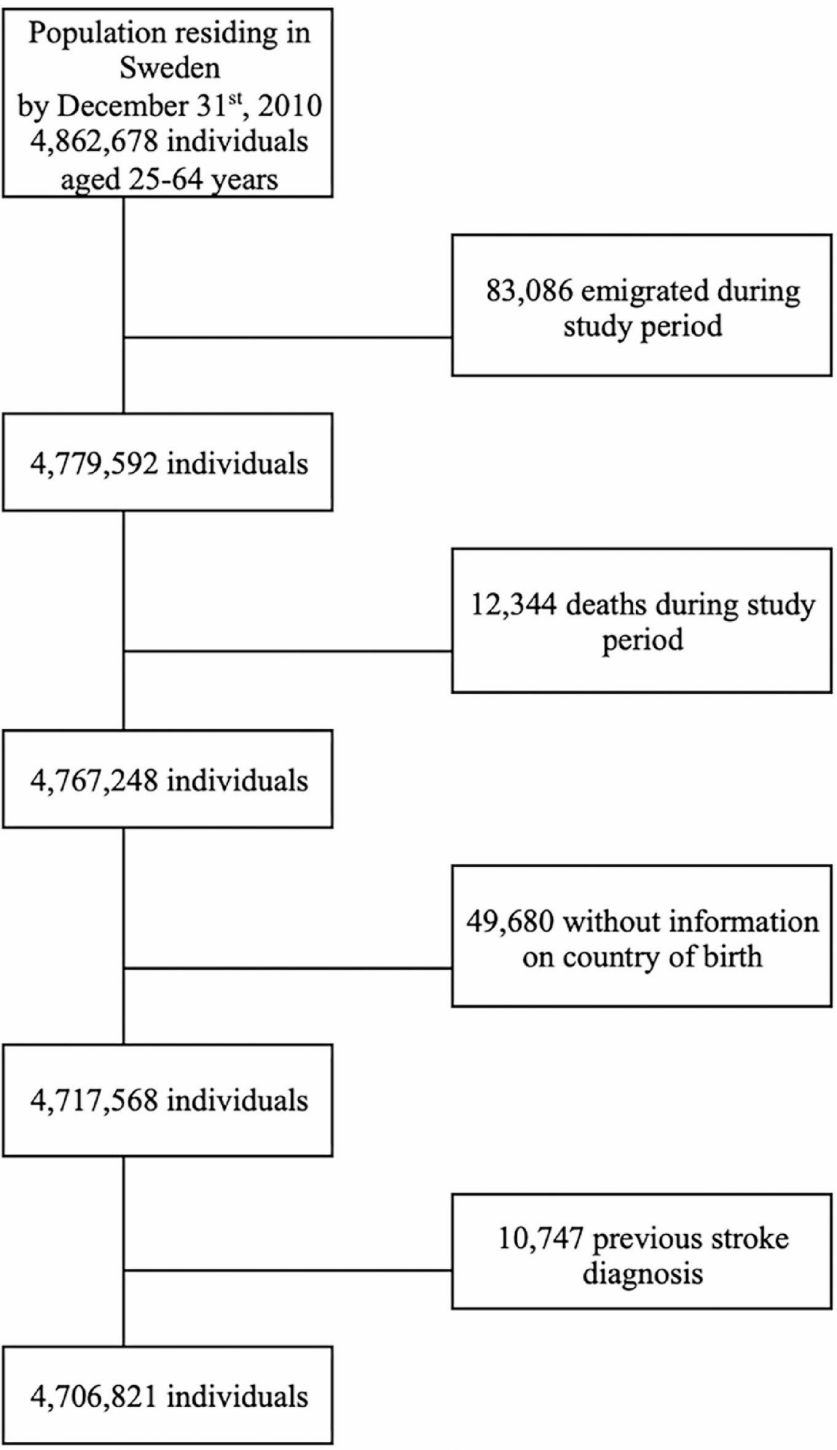
Flowchart showing the investigated population in Sweden with excluded individuals based on emigration, death, missing information, and a previous stroke diagnosis. The flowchart shows the initial population with excluded individuals that were analysed concerning the consumption of psychotropic drugs as a proxy for psychological health in association to CRPS and nerve injuries, with and without surgical treatment, in the upper or lower limbs.

Our research database consisted of the total Swedish population of 2010. From the approximately 4.9 million individuals aged 25–64 years, we excluded those who emigrated (*n* = 83,086) or died (*n* = 12,344) during the study period, those whose country of birth was not registered (*n* = 49,680) and those with a previous stroke diagnosis (*n* = 10,747), as CRPS may be secondary to the stroke^26^. The final sample consisted of around 4.7 million individuals (Fig. 1).

#### Assessment of variables

We defined the *diagnosis of CRPS* according to the International Classification of Diseases, 10th revision (ICD-10)^27^ codes (M89.0, G90.5, G90.6, and G90.7) between 2011 and 2013, which does not distinguish between upper or lower limb. Previous CRPS diagnosis was defined as having such a diagnosis the year before inclusion in the cohort.

The two types of CRPS (*type 1* = no apparent nerve lesion and *type 2* = an apparent nerve lesion, such as a nerve injury or a nerve entrapment disorder triggering the onset of CRPS), are not explicitly coded in the NPR. CRPS type 1 was defined by the mentioned International Classification of Diseases (ICD)-codes and without any apparent nerve lesion, while CRPS type 2 was defined if a concomitant *nerve lesion (including nerve entrapment disorder) or* *a previous nerve surgery for those lesions* were present one year before the CRPS diagnosis (see below). For this purpose we used specific ICD-10 codes: *nerve compression disorders*, *nerve injuries and neuroma*, and *amputations*. We included CRPS in both the upper and lower limbs because the ICD-10 codes do not distinguish between anatomical locations. See Supplemental Table 1 for a list of the specific explanations of the codes.

Previous nerve surgery related to the defined nerve lesions was identified by the presence of relevant surgical procedure codes, such as those for *nerve compression disorders*, *nerve injuries*, and *neuromas*, occurring one year prior to the baseline. See Supplemental Table 2 for a list of the specific explanations of the codes. Other specified mononeuropathies, unspecified mononeuropathy, and unspecified polyneuropathy, according to ICD-10 codes, were defined as being in the lower limb because most of them affect the lower limb.

Using the definitions above, we categorized the individuals into four categories:

- (a) General population – No CRPS and no nerve injuries or nerve entrapment disorders.
- (b) Nerve injuries - Nerve injuries and nerve entrapment disorders in the upper or lower limb without CRPS.
- (c) CRPS type 1 - CRPS without any nerve injury or nerve entrapment disorder.
- (d) CRPS type 2 - CRPS with any nerve injury or nerve entrapment disorder.

Thus, in the following presentation, we used the expression “nerve injuries” which included the variety of injuries and disorders as defined.

**Fig. 2:**
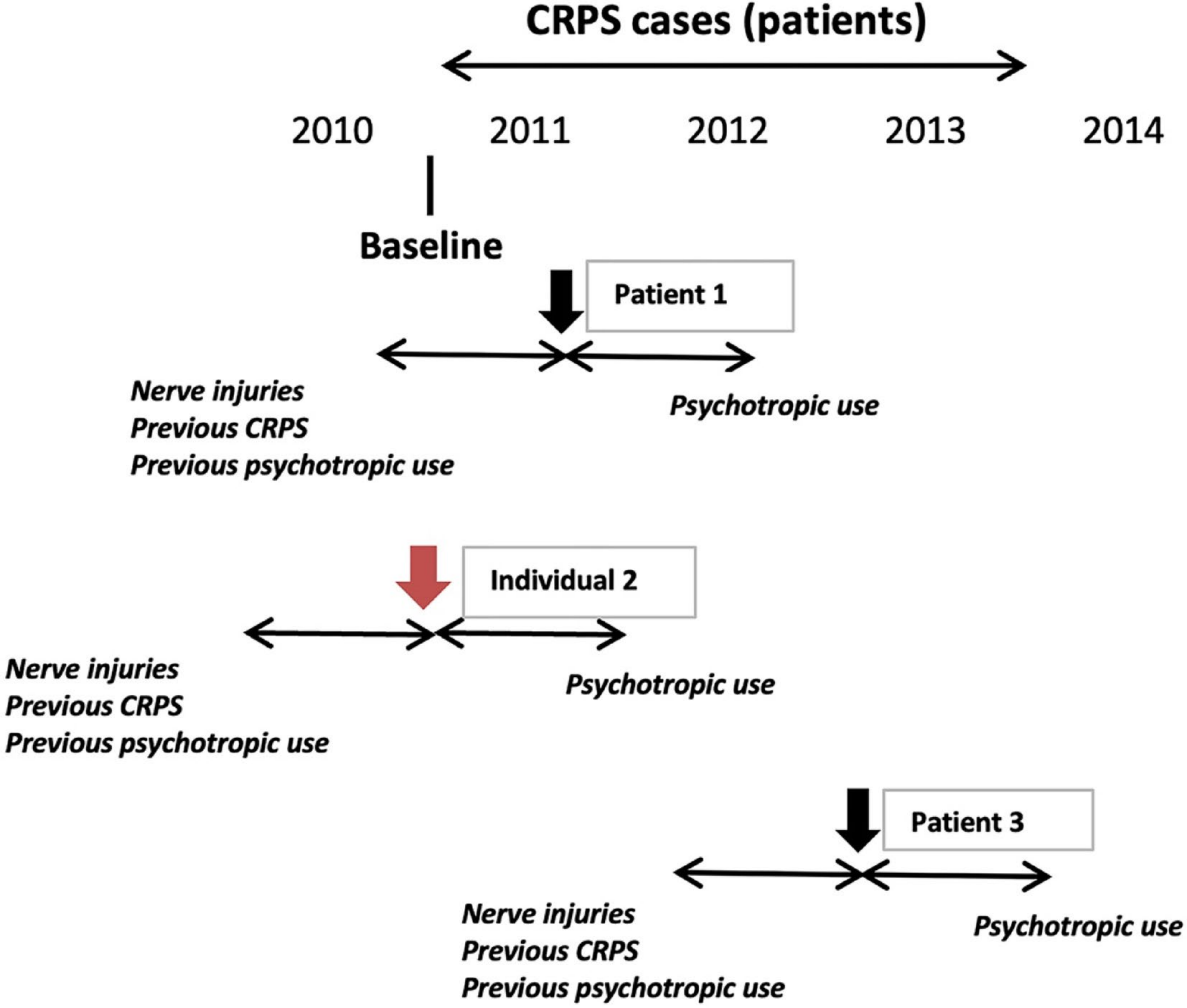
Schematic drawing of the principles of the use of psychotropic drugs in individuals, with and without Complex Regional Pain Syndrome (CRPS) or with and without a nerve injury, with an explanation of how everyone was evaluated. The cases with CRPS were sampled between January 1^st^ 2011 and December 31^st^ 2013, and each case was followed a year forward for use of psychotropic drugs and a year backward for presence of nerve injuries. The cases with CRPS type 2 were defined as having a nerve injury as defined in Methods (Supplemental Table 1 and 2; i.e., diagnostic and/or surgical codes) *one year before* the diagnosis.

We assigned an individual baseline date defined by the date of the first CRPS diagnosis between January 1^st^ 2011 and December 31^st^ 2013 or 1 st January 2011 if the individual did not have any CRPS diagnosis (category “a”). After that, we followed everyone for one year from the baseline date to ascertain their use of psychotropics (Fig. 2).

The outcome variable was the use of psychotropic drugs defined as at least one dispensation according to the Anatomical Therapeutical Chemical (ATC) classification system (code) of psycholeptics (N05), antidepressants (N06A) or psycholeptics and psychoanaleptics in combination (N06C). The following drugs with codes: N06AA09 (amitriptyline), N06AX21 (duloxetine), and N06AX16 (venlafaxine), were excluded We considered the use of psychotropics as a proxy for impaired psychological health^23–25^. The use of psychotropic drug dispensation as a proxy for impaired psychological health has been validated in several Swedish population-based studies utilizing national registry data^23–25^. To minimize misclassification due to psychotropic agents prescribed for neuropathic pain, we explicitly excluded medications with overlapping therapeutic indications - namely amitriptyline (N06AA09), duloxetine (N06AX21), and venlafaxine (N06AX16) - in accordance with national treatment guidelines for CRPS and peripheral nerve injuries in Sweden, i.e., they are interpreted instead as psychoactive drugs. Additionally, the analysis adjusted for prior psychotropic use, which helps distinguish between pre-existing psychological morbidity and new-onset distress post-CRPS diagnosis. The outline of the method is described in Fig. 2.

For instance, the CRPS **patient 1** got the CRPS diagnosis on 1 st March 2011, and then the follow-up for psychotropic drug use (i.e., the outcome) was from that point to 1 st March 2012. In this patient 1, we evaluated any nerve injury or nerve entrapment disorder as well as previous CRPS and previous use of psychotropics one year before, from 1 st March 2010 to 1 st March 2011. For the **individual 2**, *without any CRPS diagnosis* between January1^st^ 2011 and December 31^st^ 2013, we assessed nerve injury or nerve entrapment disorder as well as previous CRPS and previous use of psychotropics during the whole of 2010, and the follow-up for psychotropic drug use was observed in 2011. The CRPS **patient 3** got the CRPS diagnosis on 15 June 2012, and any nerve injury or nerve entrapment disorder, as well as previous CRPS and previous use of psychotropics, was evaluated between 15 June 2011 and 15 June 2012 while the follow-up for psychotropic drug use was assessed from 15 June 2012 to 15 June 2013.

*Previous psychotropic drug use* was defined as any dispensation of psycholeptics (N05), antidepressants (N06A), or psycholeptics and psychoanaleptics in combination (N06C) one year before the baseline (i.e., N06AA09, N06AX21, and N06AX16 were excluded because these are used as a treatment of neuropathic pain and CRPS). *Ag*e was categorized into 25-34- (reference), 35-44-, 45-54-, and 55-64-year-olds. Continuous age was included as a quadratic function in the age-stratified regression analyses. According to the register, *sex* was coded as male (reference) or female. The individuals were categorized according to their *country of birth* (COB) as native (i.e., born in Sweden (reference) or immigrant (i.e., not born in Sweden) Information on individualized disposable family *income* was obtained for the years 2000, 2005, and 2010 and we categorized into three groups by tertiles [low, medium, or high (i.e., reference) income] following an procedure described elsewhere^24,25^. Five *occupational skill* groups were used to categorize the occupational qualification level, which reflects the type of working task with their complexity (i) low, (ii) middle-low, (iii) middle-high, (iv) high, and (v) missing according to the Swedish Standard Classification of Occupations 2012^28^. See elsewhere for a detailed explanation^24,25^.

A total of 491,207 individuals (9.88%) were classified as missing as no information on occupational skill was available. The percentage of cases with no information on occupation in the different age categories was 13.67% in 25–34 years old, 8.59% in 35–44 years old, 9.39% in 45–54 years old, and 12.12% in the category 55–64 years old.

#### Statistical analyses

Since the prevalence of psychotropic use was relatively high, the association between the use of psychotropic drugs and the explanatory variables was measured by prevalence ratios (PRs) rather than odds ratios (ORs), since PRs are more accurate and interpretable in common conditions and that ORs can significantly overestimate the strength of an association with risk for misinterpretation. For this purpose, we applied Cox regression with constant time at risk^29^. Two regression models were developed. Model 1 included only information on CRPS and if the subject had a nerve injury as defined above, while model 2 added demographical and socioeconomic variables (i.e., age, sex, occupational qualification level, and country of birth) as well as previous CRPS and previous psychotropic use.

We calculated the absolute risk (AR) of use of psychotropics as the percentage of individuals using psychotropic drugs overall and in each demographic and socioeconomic stratum. Then, we calculated the absolute risk difference (ARD) by comparing AR of the evaluated groups (nerve injuries as defined, CRPS type 1 and CRPS type 2) with the AR observed among the individuals without such diagnoses in the general population. The percentages were obtained from the predicted probabilities from the regression models adjusted for age, previous use of psychotropic drugs, and previous CRPS diagnosis.

The Discriminatory Accuracy (DA) was estimated for each model by calculating the area under the receiver operating characteristic curve (AUC) with 95% confidence intervals (CI). The value of the AUC ranges from 0.5 to 1, with 1 representing perfect discrimination and 0.5 indicating no predictive accuracy^30^. Using the criteria proposed by Hosmer and Lemeshow^31^ DA was classified as absent or very weak (AUC = 0.50–0.60), poor (AUC > 0.60–⩽0.70), acceptable (AUC > 0.70– ⩽0.80) or excellent (AUC > 0.80–0.90) and outstanding (AUC > 0.90).

#### Ethics

The project was performed in accordance with relevant guidelines and regulations as well as in accordance with the declaration of Helsinki (https://www.wma.net/policies-post/wma-declaration-of-helsinki-ethical-principles-for-medical-research-involving-human-subjects/). The Regional Ethical Committee in the South of Sweden, Lund, Sweden approved the study (approval number #: 2014 − 856) and revision was performed and consent were provided by the registers´ data safety committees. Due to the retrospective nature of the study, the Regional Ethical Committee waived the need to obtain informed consent.

## Results

Table 1 suggests that the prevalence of CRPS and nerve injuries in the upper or lower limbs was very low in the population sample, ranging from 0.01% for CRPS type 2 to 0.53% for nerve injuries. Among the 4,680,834 individuals without a CRPS diagnosis or a nerve injury (i.e., “General population”), the consumption of the defined psychotropic drugs was about 15%. In contrast, the consumption among individuals diagnosed with a nerve injury in the upper or lower limb was higher, being about 25% and 30%, respectively, and this use was highest in those with CRPS type 1 and type 2 diagnoses, with about 34% and 41%, respectively.

**Table 1:** Characteristics of the 4,706,821 individuals residing in Sweden by 2010 and included in the study sample in relation to the diagnosis of nerve injuries in upper or lower limb as well as complex regional pain syndrome (CRPS) type 1 and type 2 by use of psychotropic drugs and demographical and socioeconomic factors. Values are number (n) and percentages (%).

		General population	CRPS^a^			Nerve injuries^b^		
			Type 1	Type 2	All	Upper limb	Lower limb	All^c^
*Study sample*	n	4,680,834	809	225	1,034	22,937	4,630	25,178
	%	99.45	0.02	0.01	0.02	0.49	0.10	0.53
*Psychotropic drugs*	n	693,041	276	92	368	5.807	1,390	6,596
	%	14.73	34.12	40.89	35.59	25.32	30.02	26.20
*Age (years)*								
─ 25–34	n	1,103,392	118	32	150	3,075	541	3,302
	%	23.45	14.59	14.22	14.51	13.41	11.68	13.11
─ 35–44	n	1,247,525	203	70	273	5,646	927	6,035
	%	26.51	25.09	31.11	26.40	24.62	20.02	23.97
─ 45–54	n	1,196,432	265	78	343	7,222	1,364	7,849
	%	25.42	32.76	34.67	33.17	31.49	29.46	31.17
─ 55–64	n	1,158.438	223	45	268	6,994	1,798	7,992
	%	24.62	27.56	20.00	25.92	30.49	38.83	31.74
*Women*	n	2,325,878	531	162	693	14,606	2,519	15,588
	%	49.43	65.64	72.00	67.02	63.68	54.41	61.91
*Income*								
─ Low	n	1,240,470	300	101	401	6,739	1,112	7,302
	%	26.36	37.08	44.89	38.78	29.38	24.02	29.00
─ Middle	n	1,623,387	278	79	357	8,012	1,574	8,813
	%	34.50	34.36	35.11	34.53	34.93	34.00	35.00
─ High	n	1,841,930	231	45	276	8,186	1,944	9,063
	%	39.14	28.55	20.00	26.69	35.69	41.99	36,00
*Immigrant*	n	814,460	196	66	262	3,868	704	4,227
	%	17.31	24.23	29.33	25.34	16.86	15.21	16.79
*Occupational qualification level*								
─ Low	n	265,971	76	27	103	1,888	256	2,003
	%	5.65	9.39	12.00	9.96	8.23	5.53	7.96
─ Middle-low	n	2,060,985	422	125	547	12,224	2,069	13,267
	%	43.80	52.16	55.56	52.90	53.29	44.69	52.69
─ Middle-high	n	813,647	99	19	118	3,180	801	3,511
	%	17.29	12.24	8.44	11.41	13.86	17.30	13.94
─ High	n	1,054,626	98	16	114	3,246	939	3,634
	%	22.41	12.11	7.11	11.03	14.15	20.28	14.43
─ Missing	n	510,558	114	38	152	2,399	565	2,763
	%	10.85	14.09	16.89	14.70	10.46	12.20	10.97
*Previous CRPS*	n	257	84	31	115	68	25	83
	%	0.01	10.38	13.78	11.12	0.30	0.50	0.33
*Previous psychotropic use*	n	663,248	295	76	371	5,712	1,365	6,473
	%	14.09	36.46	33.78	35.88	24.90	29.48	25.71

^a^All individuals; upper or lower limb. ^b^ Nerve injuries were defined as nerve compression disorders, nerve injuries with or without neuroma, and amputations (see Methods for details and Supplental Table 1 and 2). ^**c**^ Some patients could have had a nerve injury in both the upper and lower limb.

The percentage of individuals among the age categories in the general population was rather similar, but more individuals with CRPS diagnoses were in the middle-aged group, and more of those with nerve injuries were in the older age groups. Women were clearly overrepresented among the individuals with CRPS and nerve injury diagnoses. For instance, in the general population, 49% of the individuals were women, while in the CRPS type 1, CRPS type 2, and nerve injury groups, these values were 66%, 72%, and 62%, respectively. Individuals with low income tended to be more frequent in the groups with a CRPS diagnosis, but not in the group with nerve injuries, while the pattern was heterogeneous for individuals with high income. For instance, in the CRPS type 2 group, only 20% of the patients had high income, but this percentage was 42% in the lower limb nerve injuries and 39% in the general population. The general population’s percentage of immigrants was 17%, equal to the nerve injury group (17%) but higher in the CRPS group (25%). Individuals with low and middle-low occupational qualification levels were more represented in the CRPS and nerve injury groups than in the general population, while the opposite was true concerning individuals with high occupational qualification level. Previous CRPS was clearly overrepresented in the groups with a CRPS diagnosis, and so was previous use of psychotropic drugs. For instance, the percentage of use of psychotropics was 14% in the general population group but as much as 36% in the CRPS group and 26% among the nerve injuries.

**Fig. 3:**
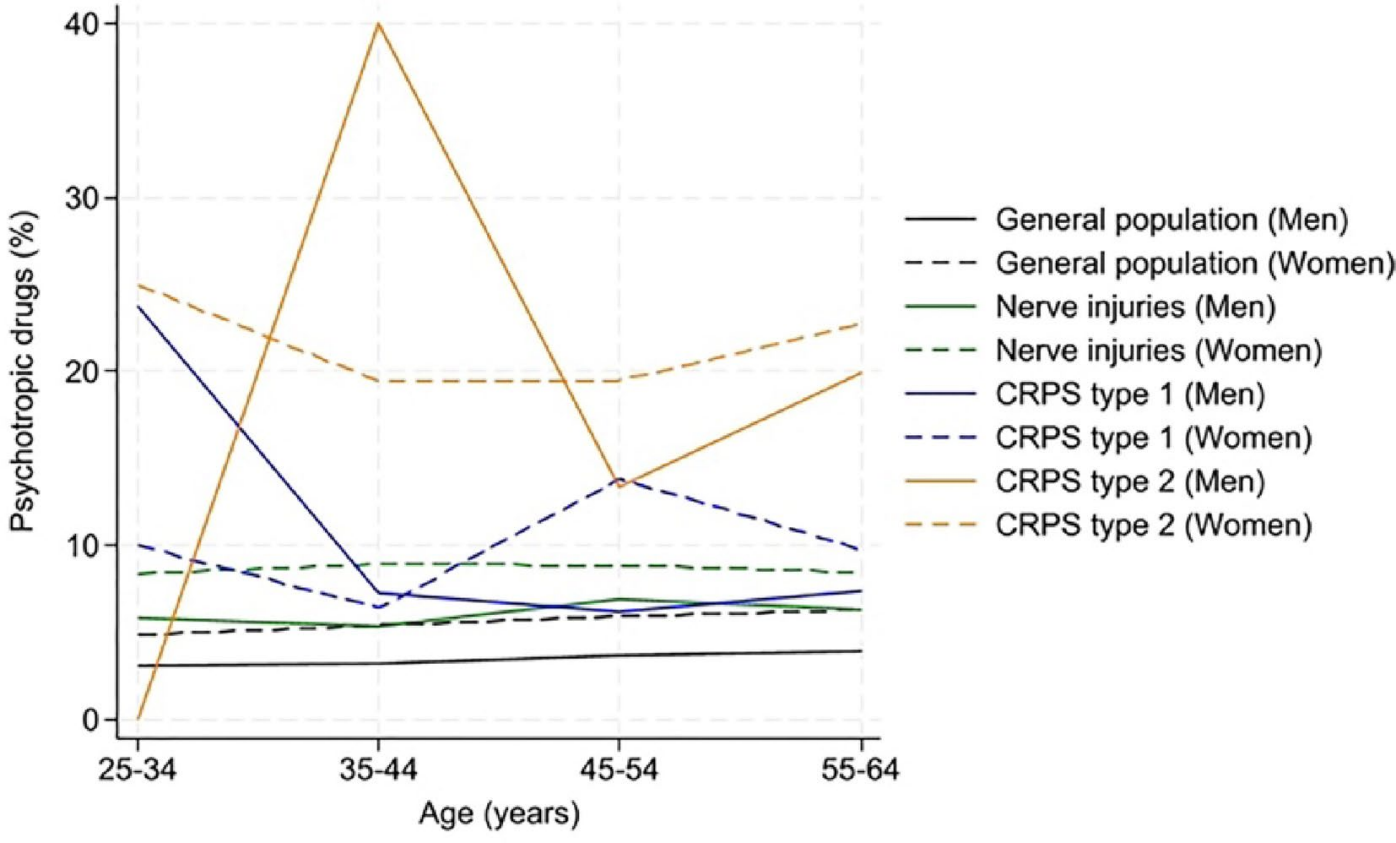
Figure 3 shows the AR of psychotropic drug use among men and women in relation to the existence of CRPS type 1 and CRPS type 2, as well as nerve injuries across age categories. The AR was highest in the groups with CRPS type 2 for both men and women, especially in 35–44-year-old men. However, in the youngest age category, the use of psychotropics was highest in men with CRPS type 1.

 Fig 3. Age and sex-specific absolute risk of use of psychotropic drugs after a diagnosis of Complex Regional Pain Syndrome (CRPS) and nerve injuries in upper or lower limb. The diagram shows the age and sex-specific absolute risk of use of psychotropic drugs during the year after a diagnosis of Complex Regional Pain Syndrome (CRPS), with (type 2) or without (type 1) a nerve injury, and nerve injuries in the upper or lower limb in 4,706,821 individuals aged 25–64 and residing in Sweden by 2010.

Table 2 shows the relative risks, expressed as PRs (95% CI), of psychotropic consumption in relation to CRPS and nerve injuries with those without such diagnoses (general population) as reference. In the unadjusted analysis (model 1), the relative risk was about two times higher in individuals with nerve injuries [PR = 1.78 (1.73–1.82)] and CRPS type 1 [PR = 2.33 (2.07–2.62)] and almost three times higher in individuals with CRPS type 2 [PR = 2.79 (2.27–3.42)]. However, those relative risks were considerably reduced when adjusting (model 2) for demographic and socioeconomic factors [nerve injuries = 1.10 (1.07–1.12); CRPS type 1 = 1.08 (1.06–1.17); CRPS type 2 = 1.36 (1.11–1.68)] as well as previous CRPS and previous psychotropic use [1.16 (0.98–1.36) and 16.09 (16.00-16.18), respectively].

**Table 2:** Prevalence ratios (PR) and 95% confidence intervals (CI) informing on the association between the existence of complex regional pain syndrome (CRPS) or nerve injuries as defined in upper or lower limbs and the use of psychotropic drugs during the year after the diagnosis in 4,706,821 individuals aged 25–64 years and residing in Sweden by 2010. Model 1 is crude, and model 2 is adjusted for demographical and socioeconomic factors (i.e., age, sex, occupational qualification level, and country of birth) as well as previous CRPS and previous psychotropic use.

	Model 1	Model 2
	PR (95% CI)	PR (95% CI)
─ General population	Reference	Reference
─ Nerve injuries	1.78 (1.73–1.82)	1.10 (1.07–1.12)
─ CRPS type 1	2.33 (2.07–2.62)	1.08 (1.06–1.17)
─ CRPS type 2	2.79 (2.27–3.42)	1.36 (1.11–1.68)
*Previous CRPS*		
─ No		Reference
─ Yes		1.16 (0.98–1.36)
*Previous psychotropic use*		
─ No		Reference
─ Yes		16.09 (16.00-16.18)
AUC	0.503	0.873

Table 3 shows the AR and ARD of using psychotropics in relation to the presence of CRPS type 1 and CRPS type 2 as well as nerve injuries with the general population (i.e., no CRPS or no nerve injury as defined) as reference. These estimations were done separately in different demographic and socioeconomic strata. In the low-income stratum, the AR of psychotropic use was 17% in the non-CRPS group, which was higher than in the middle- (AR = 15%) and high-income stratum (AR = 13%). However, we could also observe that the association between having a CRPS type 2 and the use of psychotropics was highest in the high-income stratum (ARD = 17%-units), while in the low- and middle-low-income strata, the ARD was only 7 and 8%-units, respectively. It appears that having a CRPS type 2 affects high-income individuals more as it increases the AR from 13 to 30%. The corresponding figure for CRPS type 1 was different, with a smaller ARD (3.22%-units) among high-income individuals and an even smaller ARD in individuals with nerve injuries (1.85%-units). Among the latter individuals, the ARD was higher among the low-income category (2.87%-units).

**Table 3:** Socio-economic stratified absolute risk (AR) and absolute risk difference (ARD), with 95% confidence intervals (CI) of psychotropic medication use during the year after a diagnosis of complex region pain syndrome (CRPS) and nerve injuries in upper and lower limbs in the 4,706,821 individuals aged 25–64 residing in Sweden by 2010. Values are percentages and percentage unit differences adjusted for age, previous psychotropic drugs consumption and previous CRPS diagnosis.

	General population				Nerve injuries			CRPS						
								Type 1				Type 2		
	*N*		AR	ARD	*N*	AR	ARD (95% CI)	*N*		AR	ARD (95% CI)	*N*	AR	ARD (95% CI)
**Income**														
Low	1,233,269		17.07	Ref	7,201	19.94	2.87 (2.25–3.50)		300	18.40	1.34 (−1.39-4,06)	101	24.39	7.33 (1.16–13.49)
Middle	1,614,653		15.05	Ref	8,734	16.98	1.92 (1.41–2.43)		278	15.92	0.87 (−1.65-3.38)	79	23.19	8.13 (1.14–15.13)
High	1,832,912		12.84	Ref	9,018	14.98	1.85 (1.37–2.33)		231	16.05	3.22 (0.10–6.33)	45	30.09	17.25 (4.72–29.78)
**Occupational status**														
Low		263,995	16.49	Ref	1,976	18.60	2.12 (0.96–3.27)		76	18.28	1.79 (−4.01-7.59)	27	20.50	4.01 (−5.98-14.00)
Middle-Low		2,047,843	14.25	Ref	13,142	16.29	2.04 (1.62–2.46)		422	15.85	1.60 (−0.55-8.52)	125	18.14	3.89 (−0.74-8.52)
Middle-high		810,155	12.50	Ref	3,492	14.51	2.02 (1.24–2.79)		99	13.05	0.56 (−3.22-4.33)	19	47.15	34.65 (10.52–58.78)
High		1,051,008	11.94	Ref	3,618	14.25	2.31 (1.54–3.08)		98	14.16	2.22 (−2.28-6.72)	16	36.98	25.03 (3.73–46.33)
Missing		507,833	24.95	Ref	2,725	27.79	2.84 (1.81–3.87)		114	28.13	3.18 (−1.93-8.29)	38	39.55	14.60 (2.12–27.08)
**Sex**														
Men		2,370,382	11.06	Ref	9,527	12.93	1.87 (1.44–2.31)		278	13.46	2.41 (0.03–4.78)	63	20.54	9.49 (1.20-17.78)
Women		2,310,452	18.46	Ref	15,426	20.64	2.18 (1.75–2.60)		531	19.44	0.97 (−1.18-3.12)	162	27.63	9.16 (3.94–14.39)
**Country of birth**														
Native		3,870,535	14.79	Ref	20,792	16.66	1.87 (1.54–2.19)		613	15.20	0.41 (−1.41-2.07)	159	22.93	8.14 (3.24–13.04)
Immigrant		810,299	14.36	Ref	4,161	18.47	4.10 (3.27–4.94)		196	21.09	6.73 (2.59–10.88)	66	28.57	14.21 (4.57–23.85)
**Age**														
25–34		1,100,122	9.94	Ref	3,270	19.71	9.77 (8.41–11.13)		118	35.32	25.38 (16.78–33.98)	32	43.60	33.66 (16.50-50.82)
35–44		1,241,560	12.79	Ref	5,965	22.79	10.09 (9.03–11.16)		203	36.10	23.40 (16.81–29.99)	70	41.38	28.67 (17.15–40.20)
45–54		1,188,661	16.17	Ref	7,771	27.28	11.11 (10.12–12.10)		265	32.40	16.23 (10.60-21.86)	78	32.95	16.78 (6.38–27.17)
55–64		1,150,491	19.75	Ref	7,947	29.64	9.89 (8.88–10.90)		223	33.30	13.56 (7.37–19.75)	45	51.60	31.85 (17.26–46.44)

Ref = reference.

The ARs varied between 12 and 16% concerning occupational status, with a slight increase from high to low. A similar pattern with slightly higher ARs (14–19%) was observed among individuals with nerve injuries, resulting in ARDs just above 2%-units. A similar pattern was also observed among individuals with CRPS type 1 (ARs 13–18% and ARDs 0.6–2.2%%-units). In contrast, the ARs were higher in individuals with CRPS type 2, particularly among those with middle-high (ARD 35%) and high (25%) occupational status. Thus, having a CRPS type 2 affects those with middle-high and high occupational status more than those with low occupational status.

Table 3 shows that women in the general population have a higher risk (AR = 18%) of using psychotropics than men (AR = 11%). However, the association between having a CRPS or nerve injuries and the use of psychotropic drugs within the stratum of men and within the stratum of women were rather similar, especially for the CRPS type 2 group, but was high for both men and women with CRPS type 2 (around 9%-units). Thus, to have such diagnoses similarly affects men and women.

We could also see that in the general population, immigrants have a similar risk of using psychotropics (AR = 14%) than natives (AR = 15%). However, the association between nerve injuries, CRPS type 1 and CRPS type 2, and the use of psychotropics was clearly higher in the immigrant stratum than in the native stratum. That is ARD = 4 versus 2%-units, 7 versus 0.4%-units, and 14 versus 8%-units, respectively. It appears that having such diagnoses affects more immigrants than natives.

The AR increased with age (from 10 up to 20% from the lowest to the highest age group) among the age groups. The ARs were higher among the individuals with nerve injuries and with the same increase with age groups, resulting in ARDs approximately at the same level across age groups (10–11%). Furthermore, the ARs were much higher in the individuals with CRPS type 1 (32–36% among age categories) and particularly with CRPS type 2 (33–52%), resulting in ARDs among these groups of 14–25%-units and 17–34%-units, respectively, with a variation among age categories. Thus, having a CRPS type 1 diagnosis affects younger individuals (25–34 and 35–44 years), and those with CRPS type 2 were not only affected among the two lower age categories (25–34 and 35–44 years), but also affected in the highest age category (55–64 years).

## Discussion

The consumption of psychotropic drugs in the general Swedish population without a diagnosis of CRPS or a nerve injury/disorder was around 15%, which corresponds well with the prevalence of use of those psychotropic drugs in Sweden according to the National Board of Health and Welfare (/https://sdb.socialstyrelsen.se/if_lak/val.aspx). These figures mirror the prevalence of impaired psychological health according to our definition^24^. However, psychotropic drug-use was higher. i.e., 26%. in those with a nerve injury in the upper or lower limb, and even higher, i.e., 36%, in individuals with a diagnosis of CRPS in the upper or lower limb, particularly in individuals with CRPS type 2, i.e., those with an associated nerve injury, suggesting that psychological health is linked, without causation^22^ to CRPS^1,13–20^.

Furthermore, compared to the general population, the unadjusted relative risk for consumption was about two times higher in patients with nerve injuries and CRPS type 1 and almost three times higher in patients with CRPS type 2. As expected, those relative risks became considerably reduced (PRs around 1.10 and 1.4 for nerve injuries/CRPS type 1 and CRPS type 2, respectively; thus, same risk relation), when adjusting for the demographic and socioeconomic factors age, sex, occupational qualification level, and country of birth. However, those factors are not clearly confounders as CRPS diagnoses may be mediators in the causal pathway leading to impaired psychological health or distress in individuals with low socioeconomic positions.

CRPS can affect the upper or lower limb, and the ICD codes are the same regardless of the limb affected. Our study is innovative as it not only investigates the risk of the use of psychotropic drugs related to CRPS diagnoses in the whole population but also how this risk is modified in different demographic and socioeconomic strata. Such information is often hidden behind average population risks. For instance, women have a higher risk of using psychotropic drugs than men, but the influence of CRPS or nerve injuries on the use of psychotropic drugs was similar within both strata. CRPS is more frequently observed in the upper limb and with an overrepresentation of women^4,32,33^ which is in accordance with our earlier study consisting of around 70% of women with CRPS type^19,20^ as well as that distal radius fractures are common among women^1^. This finding may be related to the types of included nerve injuries and nerve entrapment disorders, where women dominate. The relation between CRPS type 1 and CRPS type 2 was 3.5:1, which is in accordance with other studies^4,32^; i.e., the present database is valid.

It is known that immigrants have a lower risk of using psychotropic drugs than natives, which may be mainly attributed to insufficient access to health care^34–37^. However, the association between nerve injuries, CRPS type 1 and CRPS type 2, and use of psychotropics was certainly higher in the immigrant stratum than in the native stratum (higher ARDs for CRPS type 2 for both natives, 8%-units, and immigrants, 14%-units). That is, suffering from such diagnoses might affect more immigrants than natives, which should be further investigated. Previous psychotropic drug consumption was highly associated [16.09 (16.00-16.18)] with continuous consumption of the drugs, which is in line with opioid consumption in surgically treated neuroma patients, although it is reported to be reduced in such surgically treated patients^38^.

Individuals with CRPS generally have a poor quality of life, which differs from individuals with other chronic pain conditions. Based on in-depth interviews, individuals with CRPS have described it as a war-like experience (i.e., “dealing with the unknown enemy”, “building an armory against a moving target”, “battles within the war”, “developing battle plans with allies” and “warrior or prisoner of war”^39^). Factors, such as psychological behaviour, depression, preoperative psychological distress, or an increased pain level, are not predictive for the development of CRPS^2,10,21^ but anxiety and depression, on the other hand, are linked to CRPS^13,14^. In addition, individuals with CRPS type 1 are not psychologically different from the general population^40,41^. One may argue that psychological factors and psychiatric conditions should not be interpreted as causing the condition but rather are a result of the condition leading to psychological stress^1,15–18^. Our recent data suggest that both aspects may be relevant^19,20^. In this context, one may stress that individuals with CRPS may suffer from pain, sleeping problems, limitations in daily activities, anxiety, and depression, which may have an impact on their life^20^. Such patients may also have a low sense of coherence and high pain catastrophizing scores associated with a worse outcome, impacting the development of CRPS^20,42,43^. One previous large study showed an association between depression and CRPS, but the cross-sectional design makes it impossible to draw any conclusions on causality^44^.

Women were at greater risk of consuming psychotropic drugs than men, indicating that women with CRPS may suffer more psychological distress^4^ than men with CRPS. However, the ARDs were similar between men and women in the three groups of individuals with the diagnoses although being lower for nerve injuries and CRPS type 1 (ARDs < 2.41%-units) and higher for CRPS type 2 (ARDs 9.2–9.5%-units). One other study found contradictory results, with men reporting more depression and more kinesiophobia than women with CRPS^45^. This might be explained by that men reported worse coping strategies and were less likely to seek support. Hence, women may be more prone to seek help for their depressive symptoms and, therefore, more likely to be prescribed medication or more likely to receive such help when seeking care, which may mirror the findings concerning occupational and income levels (see below). Central sensitisation, i.e., amplification of neural signaling in nociceptive spinal and trigeminal neurons, can induce facilitation of pain hypersensitivity; a feature of CRPS together with a dysregulated sensory processing^4^. Data from functional MRI studies also suggest several different alterations in the brain related to such findings^4^.

The present data showed a higher consumption of psychotropic drugs among individuals with CRPS type 1 compared to the general population, even when adjusted for the demographical and socioeconomic factors. Additionally, there was a higher risk for high consumption of psychotropic drugs in CRPS type 2, i.e., in those with an associated nerve injury. The risk of psychotropic consumption among the individuals with injury(ies) to peripheral nerve(s) was similar to CRPS type 1 but lower than observed for the individuals with CRPS type 2. This suggests that the nerve injury per se is relevant for psychological health or psychological distress but with an additive effect of the CRPS diagnosis. Thus, CRPS with an associated nerve injury presented the highest risk for psychotropic drug use, exceeding that of individuals with treated nerve entrapment disorders^24^indicating that the type of nerve injury may also be relevant in this context. Both the peripheral and the central sensitization of the nervous system in CRPS might also affect the risk of psychotropic drug consumption^46^.

The occupational qualification levels were also associated with a risk for the use of psychotropic drugs and nerve injury/CRPS with an especially higher risk among those with high and middle high levels (ARDs 25–35%-units) for CRPS type 2. The ARDs were much lower for the other occupational levels and nerve injuries or CRPS type 1 (ARDs < 2.31%-units). In accordance, regarding income levels, the highest association was observed in the high-income stratum (ARD = 17%-units) between having CRPS type 2 and the use of psychotropics, while ARDs were much lower in the low- and middle-low-income strata (7 and 8%-units). This observation that CRPS type 2 affects high-income individuals more (AR increase 13–30%) warrants further investigation because the ARDs were much lower for nerve injuries and CRPS type 1 (< 3.22%-units). Higher ARDs (34 and 32%-units) were particularly observed in individuals with CRPS type 2 at age 25–34 and 55–64 years. In contrast to the findings of CRPS type 2, low occupational or income levels have an impact on use of psychotropic and psychoactive drugs in other nerve entrapment disorders in the upper limb^24,25^. However, still, our data propose that socioeconomic factors, as income level, occupational status, country of birth, as well as sex and age of the individuals influence the consumption in accordance with observations of high risk of consumption of opioids and gabapentoid drugs as well as psychotropic drugs in patients with nerve entrapment disorders^24,25^. Disability and pain severity are more intensely associated with psychological factors in CRPS than in other diagnoses with pain, i.e., low back pain^47,48^. A history of previous CRPS was also associated with a higher risk, which is plausible since there, for different reasons, including genetic heredity, is a propensity in some individuals to develop CRPS^16,49–51^. The reason why a previously surgically treated nerve injury in individuals (see Supplemental Tables 1 and 2) is not associated with the use of psychotropic drugs is more obscure but may hypothetically be related to less risk of having a permanent nerve injury or prevention of formation of a neuroma^38^.

This study has several *limitations* that should be considered when interpreting the findings.

*Classification of CRPS: *The primary limitation is the inability to distinguish between CRPS affecting the upper versus lower limbs. This limitation arises from the structure of ICD-10 coding, which does not provide site-specific classification for CRPS. The updated ICD-11 system allows for such differentiation and may enable more detailed analyses in future studies.

*Incomplete Exclusion of Rare Causes: *Although we excluded CRPS cases potentially caused by stroke, it was not possible to comprehensively rule out all less frequent etiologies. This may introduce heterogeneity into the study population.

*Proxy Measures of Psychological Health: *Psychological health was approximated using registry-based data on dispensed psychotropic medications. This approach has inherent limitations as detailed clinical data on CRPS severity, concomitant injuries (including nerve damage), or treatment outcomes were not available.

Also, misclassification may have occurred, as dispensation records do not confirm medication adherence or actual use.

*Residual Confounding and Confounding by Indication: *Despite adjustments, residual confounding may persist due to unmeasured factors, such as comorbidities, unrecorded psychological conditions, and variability in healthcare access. Confounding by indication is particularly relevant in this context: psychotropic drugs may be prescribed not only for psychological conditions (e.g., depression, anxiety) but also for chronic pain, insomnia, or other off-label indications. This overlap may inflate the observed association between CRPS and psychotropic drug use. To mitigate this, we excluded medications primarily indicated for neuropathic pain and adjusted for prior psychotropic drug use. However, complete disentanglement of psychological distress from pharmacological pain management is inherently challenging in register-based studies.

*Sensitivity Analyses: *A sensitivity analysis that included previously excluded medications used for neuropathic pain (ATC codes N06AA09, N06AX21, and N06AX16, as per national guidelines) yielded consistent results: PR for nerve injuries: 1.09 (95% CI, 1.07–1.12), PR for CRPS type 1: 1.27 (95% CI, 1.15–1.40), PR for CRPS type 2: 1.41 (95% CI, 1.18–1.69). These findings support the robustness of the primary results.

*Definition of Exposure: *Psychotropic drug exposure was defined as at least one dispensation during the one-year follow-up period. We did not conduct sensitivity analyses to distinguish acute from chronic use, nor did we apply a washout period as used in some pharmacoepidemiologic research. These decisions reflect our aim to capture broad, population-level patterns of psychotropic drug use following CRPS diagnosis, the limited clinical detail in the registry data regarding treatment duration and indication, and consistency with previous studies^23–25^. Nonetheless, potential misclassification of exposure timing remains a consideration and should be addressed in future research with more detailed data.

The *strength* is that we included data from a substantial part of the Swedish population, which allowed us to distinguish the individuals with nerve injuries in the upper or lower limbs from the ones with CRPS type 1 and CRPS type 2. Ideally, the association between CRPS diagnoses and the use of psychotropic drugs should be investigated in individuals neither with a previous CRPS diagnosis nor with previous psychotropic use. However, we adjusted for those variables in the model as this restriction was impossible to implement because the number of individuals with specific types of CRPS was relatively small. In any case, our results implied a higher risk of psychotropic drug use in patients with CRPS, particularly in those with an associated nerve injury (CRPS type 2), and in those with nerve injuries, and this information seems clinically relevant.

## Conclusions

Individuals with CRPS, or nerve injuries without CRPS, in the upper or lower limbs have a high risk of using psychotropic drugs as a proxy for psychological health problems or psychological distress compared to the general population and with an impact of some demographic and socioeconomic factors. The risk is even higher in CRPS with a nerve injury (CRPS type 2). Careful treatment of individuals with nerve injuries, with or without CRPS, is crucial. An individual’s psychological health, also reflecting medication adherence or consumption of the prescribed drugs, should be considered. The new CRPS classification in ICD-11, with linkage to other registers, may enable an improved analysis of risk factors, consumption of different medications and the impact of a concomitant nerve injury and analysis of confounders in the future.

## Electronic supplementary material

Below is the link to the electronic supplementary material.

Supplementary Material 1

Supplementary Material 2

## Data Availability

The data that support the findings of this study are available by request (and construction) to the Swedish National Board of Health and Welfare and Statistics Sweden after approval of the research project by the Ethical Committee and by the data safety committees of the Swedish Authorities but restrictions apply to the availability of these data, which were used under license for the current study, and so are not publicly available. Data are not available from the corresponding authors upon request for ethical and/or legal reasons due to compromising patient privacy but permission has to be requested from The National Ethical Committee via the homepage of Etikprövningsmyndigheten in Sweden (etikprovningsmyndigheten.se). A study also needs to be performed in collaboration with Swedish researchers.
