# Supplementary material for: Complex regional pain syndrome and use of psychotropic drugs as a proxy for psychological health
Source: Sci Rep. 2025 Jul 10;15:24963. doi: 10.1038/s41598-025-09701-9 (PMC12246038; doi:10.1038/s41598-025-09701-9)
Supplement: Supplementary file 1 — Supplementary Material 1 [file 41598_2025_9701_MOESM1_ESM.pdf]

Supplemental table 1 Nerve injury diagnoses

Nerve compression disorders

G56.0	Carpal tunnel syndrome
G56.1	Other lesions of median nerve
G56.2	Lesion of ulnar nerve
G56.3	Lesion of radial nerve
G56.8	Other specified mononeuropathies of upper limb
G56.9	Unspecified mononeuropathy of upper limb
G57.5	Tarsal tunnel syndrome
G57.8	Other specified mononeuropathies of lower limb
G57.9	Unspecified mononeuropathy of lower limb
O26.8D	Carpal tunnel syndrome - the specified pregnancy-related condition

Nerve injuries and neuroma

G57.0	Lesion of sciatic nerve
G57.1	Meralgia paresthetica
G57.2	Lesion of femoral nerve
G57.3	Lesion of lateral popliteal nerve
G57.4	Lesion of medial popliteal nerve
G57.6	Lesion of plantar nerve
G57.7	Causalgia of lower limb
G58.8	Other specified mononeuropathies
G58.9	Mononeuropathy. unspecified
G62.9	Polyneuropathy. unspecified
S44.0	Injury of ulnar nerve at upper arm level
S44.1	Injury of median nerve at upper arm level

S44.2	Injury of radial nerve at upper arm level
S44.3	Injury of axillary nerve
S44.4	Injury of musculocutaneous nerve
S44.5	Injury of cutaneous sensory nerve at shoulder and upper arm level
S44.7	Injury of multiple nerves at shoulder and upper arm level
S44.8	Injury of other specified nerves at shoulder and upper arm level
S44.9	Injury of unspecified nerve at shoulder and upper arm level
S54.0	Injury of ulnar nerve at forearm level
S54.1	Injury of median nerve at forearm level
S54.2	Injury of radial nerve at forearm level
S54.3	Injury of cutaneous sensory nerve at forearm level
S54.7	Injury of multiple nerves at forearm level
S54.8	Injury of other specified nerve at forearm level
S54.9	Injury of unspecified nerve at forearm level
S64.0	Injury of ulnar nerve at wrist and hand level
S64.1	Injury of median nerve at wrist and hand level
S64.2	Injury of radial nerve at wrist and hand level
S64.3	Injury of digital nerve of thumb
S64.4	Injury of digital nerve of other and unspecified finger
S64.7	Injury of multiple nerves at wrist and hand level
S64.8	Injury of other specified nerves at wrist and hand level
S64.9	Injury of unspecified nerve at wrist and hand level
S74.0	Injury of sciatic nerve at hip and thigh level
S74.1	Injury of femoral nerve at hip and thigh level
S74.2	Injury of cutaneous sensory nerve at hip and thigh level
S74.7	Injury of multiple nerves at hip and thigh level
S74.8	Injury of other specified nerves at hip and thigh level

S74.9	Injury of unspecified nerves at hip and thigh level
S84.0	Injury of tibial nerve at lower leg level
S84.1	Injury of peroneal nerve at lower leg level
S84.2	Injury of cutaneous sensory nerve at lower leg level
S84.7	Injury of multiple nerves at lower leg level
S84.8	Injury of other specified nerves at lower leg level
S84.9	Injury of unspecified nerves at lower leg level
S94.0	Injury of lateral plantar nerve
S94.1	Injury of medial plantar nerve
S94.2	Injury of deep peroneal nerve at ankle and foot level
S94.3	Injury of cutaneous sensory nerve at ankle and foot level
S94.7	Injury of multiple nerves at ankle and foot level
S94.8	Injury of other specified nerves at ankle and foot level
S94.9	Injury of unnerves at ankle and foot level
T87.3	Neuroma in amputation stump
T87.3B	Neuroma in amputation stump shoulder and upper arm
T87.3C	Neuroma in amputation stump elbow/forearm
T87.3D	Neuroma in amputation stump wrist and hand level
T87.3F	Neuroma in amputation stump hip and thigh
T87.3G	Neuroma in amputation stump knee/lower leg
T87.3H	Neuroma in amputation stump ankle and foot
T87.3X	Neuroma in amputation stump unspecified location
T92.4	Sequelae of injury of nerve of upper limb
T92.6	Sequelae of crushing injury or traumatic amputation of upper limb
T93.4	Sequelae of injury of nerve of lower limb
T93.6	Sequelae of crush injury and of traumatic amputation of lower limb

T94.0	Sequelae of injuries involving multiple body regions

Amputations

G54.6	Phantom limb syndrome with pain
S48.0	Traumatic amputation at shoulder joint
S48.1	Traumatic amputation at level between shoulder and elbow
S48.9	Traumatic amputation at shoulder and upper arm at unspecified level
S58.0	Traumatic amputation at elbow level
S58.1	Traumatic amputation at level between elbow and wrist
S58.9	Traumatic amputation of forearm at unspecified level
S68.0	Traumatic amputation of thumb (complete or partial)
S68.1	Traumatic amputation of another single finger (complete or partial)
S68.2	Traumatic amputation of two or more fingers alone (complete)(partial)
S68.3	Combined traumatic amputation of (part of) finger(s) with other parts of wrist and hand
S68.4	Traumatic amputation of hand at wrist level
S68.8	Traumatic amputation other specified parts of wrist or hand
S68.9	Traumatic amputation other of wrist or hand at unspecified level
S69.7	Multiple injuries at wrist or hand
T05.0	Traumatic amputation of both hands
T05.1	Traumatic amputation of one hand and other arm [any level. except hand]
T05.2	Traumatic amputation of both arms [any level]
T05.3	Traumatic amputation of both feet [any level]

T05.4	Traumatic amputation of one foot and other leg [any level. except foot]
T05.9	Multiple traumatic amputations. unspecified

Supplemental table 2 Surgical procedures

Nerve compression disorders

ACC43	Transposition of peripheral nerve – ulnar nerve
ACC51	Decompression av peripheral nerve – median nerve
ACC52	Decompression av peripheral nerve – radial nerve
ACC53	Decompression av peripheral nerve - ulnar nerve
ACC54	Decompression av peripheral nerve – peroneal nerve
ACC55	Decompression av peripheral nerve – tibial nerve
ACC56	Decompression av peripheral nerve – sciatic nerve
ACC59	Decompression av peripheral nerve – other or unspecified nerve

Nerve injuries and neuroma

ACA12	Exploration of peripheral nerve – radial nerve
ACA13	Exploration of peripheral nerve – ulnar nerve
ACA14	Exploration of peripheral nerve – peroneal nerve
ACA15	Exploration of peripheral nerve – tibial nerve
ACA16	Exploration of peripheral nerve – sciatic nerve
ACA19	Exploration of peripheral nerve – other or unspecified nerve
ACB21	Suture of peripheral nerve – median nerve
ACB22	Suture of peripheral nerve – radial nerve
ACB23	Suture of peripheral nerve – ulnar nerve
ACB24	Suture of peripheral nerve – peroneal nerve
ACB25	Suture of peripheral nerve – tibial nerve
ACB26	Suture of peripheral nerve – sciatic nerve

ACB29	Suture of peripheral nerve – other or unspecified nerve
ACC12	Transection of peripheral nerve – radial nerve
ACC19	Transection of peripheral nerve - other or unspecified nerve
ACC21	Reconstruction av peripheral nerve – median nerve
ACC22	Reconstruction av peripheral nerve – radial nerve
ACC23	Reconstruction av peripheral nerve – ulnar nerve
ACC24	Reconstruction av peripheral nerve – peroneal nerve
ACC25	Reconstruction av peripheral nerve – tibial nerve
ACC26	Reconstruction av peripheral nerve – sciatic nerve
ACC29	Reconstruction av peripheral nerve - other or unspecified nerve
ACC42	Transposition of peripheral nerve – radial nerve
ACC49	Transposition of peripheral nerve - other or unspecified nerve
ZZK00	Nerve graft
