# Supplementary material for: Complex regional pain syndrome and use of psychotropic drugs as a proxy for psychological health
Source: Sci Rep. 2025 Jul 10;15:24963. doi: 10.1038/s41598-025-09701-9 (PMC12246038; doi:10.1038/s41598-025-09701-9)
Supplement: Supplementary file 2 — Supplementary Material 2 [file 41598_2025_9701_MOESM2_ESM.docx]

**Supplemental table 1 Nerve injury diagnoses**

**Nerve compression disorders**

G56.0 Carpal tunnel syndrome

G56.1 Other lesions of median nerve

G56.2 Lesion of ulnar nerve

G56.3 Lesion of radial nerve

G56.8 Other specified mononeuropathies of upper limb

G56.9 Unspecified mononeuropathy of upper limb

G57.5 Tarsal tunnel syndrome

G57.8 Other specified mononeuropathies of lower limb

G57.9 Unspecified mononeuropathy of lower limb

O26.8D Carpal tunnel syndrome - the specified pregnancy-related condition

**Nerve injuries and neuroma**

G57.0 Lesion of sciatic nerve

G57.1 Meralgia paresthetica

G57.2 Lesion of femoral nerve

G57.3 Lesion of lateral popliteal nerve

G57.4 Lesion of medial popliteal nerve

G57.6 Lesion of plantar nerve

G57.7 Causalgia of lower limb

G58.8 Other specified mononeuropathies

G58.9 Mononeuropathy. unspecified

G62.9 Polyneuropathy. unspecified

S44.0 Injury of ulnar nerve at upper arm level

S44.1 Injury of median nerve at upper arm level

S44.2 Injury of radial nerve at upper arm level

S44.3 Injury of axillary nerve

S44.4 Injury of musculocutaneous nerve

S44.5 Injury of cutaneous sensory nerve at shoulder and upper arm level

S44.7 Injury of multiple nerves at shoulder and upper arm level

S44.8 Injury of other specified nerves at shoulder and upper arm level

S44.9 Injury of unspecified nerve at shoulder and upper arm level

S54.0 Injury of ulnar nerve at forearm level

S54.1 Injury of median nerve at forearm level

S54.2 Injury of radial nerve at forearm level

S54.3 Injury of cutaneous sensory nerve at forearm level

S54.7 Injury of multiple nerves at forearm level

S54.8 Injury of other specified nerve at forearm level

S54.9 Injury of unspecified nerve at forearm level

S64.0 Injury of ulnar nerve at wrist and hand level

S64.1 Injury of median nerve at wrist and hand level

S64.2 Injury of radial nerve at wrist and hand level

S64.3 Injury of digital nerve of thumb

S64.4 Injury of digital nerve of other and unspecified finger

S64.7 Injury of multiple nerves at wrist and hand level

S64.8 Injury of other specified nerves at wrist and hand level

S64.9 Injury of unspecified nerve at wrist and hand level

S74.0 Injury of sciatic nerve at hip and thigh level

S74.1 Injury of femoral nerve at hip and thigh level

S74.2 Injury of cutaneous sensory nerve at hip and thigh level

S74.7 Injury of multiple nerves at hip and thigh level

S74.8 Injury of other specified nerves at hip and thigh level

S74.9 Injury of unspecified nerves at hip and thigh level

S84.0 Injury of tibial nerve at lower leg level

S84.1 Injury of peroneal nerve at lower leg level

S84.2 Injury of cutaneous sensory nerve at lower leg level

S84.7 Injury of multiple nerves at lower leg level

S84.8 Injury of other specified nerves at lower leg level

S84.9 Injury of unspecified nerves at lower leg level

S94.0 Injury of lateral plantar nerve

S94.1 Injury of medial plantar nerve

S94.2 Injury of deep peroneal nerve at ankle and foot level

S94.3 Injury of cutaneous sensory nerve at ankle and foot level

S94.7 Injury of multiple nerves at ankle and foot level

S94.8 Injury of other specified nerves at ankle and foot level

S94.9 Injury of unnerves at ankle and foot level

T87.3 Neuroma in amputation stump

T87.3B Neuroma in amputation stump shoulder and upper arm

T87.3C Neuroma in amputation stump elbow/forearm

T87.3D Neuroma in amputation stump wrist and hand level

T87.3F Neuroma in amputation stump hip and thigh

T87.3G Neuroma in amputation stump knee/lower leg

T87.3H Neuroma in amputation stump ankle and foot

T87.3X Neuroma in amputation stump unspecified location

T92.4 Sequelae of injury of nerve of upper limb

T92.6 [Sequelae of crushing injury or traumatic amputation of upper limb](https://gesund.bund.de/en/icd-code-search/t92-6)

T93.4 [Sequelae of injury of nerve of lower limb](https://gesund.bund.de/en/icd-code-search/t93-4)

T93.6 Sequelae of crush injury and of traumatic amputation of lower limb

T94.0 [Sequelae of injuries involving multiple body regions](https://gesund.bund.de/en/icd-code-search/t94-0)

**Amputations**

G54.6 Phantom limb syndrome with pain

S48.0 Traumatic amputation at shoulder joint

S48.1 Traumatic amputation at level between shoulder and elbow

S48.9 Traumatic amputation at shoulder and upper arm at unspecified level

S58.0 Traumatic amputation at elbow level

S58.1 Traumatic amputation at level between elbow and wrist

S58.9 Traumatic amputation of forearm at unspecified level

S68.0 Traumatic amputation of thumb (complete or partial)

S68.1 Traumatic amputation of another single finger (complete or partial)

S68.2 Traumatic amputation of two or more fingers alone (complete)(partial)

S68.3 Combined traumatic amputation of (part of) finger(s) with other parts of wrist and hand

S68.4 Traumatic amputation of hand at wrist level

S68.8 Traumatic amputation other specified parts of wrist or hand

S68.9 Traumatic amputation other of wrist or hand at unspecified level

S69.7 Multiple injuries at wrist or hand

T05.0 Traumatic amputation of both hands

T05.1 [Traumatic amputation of one hand and other arm [any level. except hand]](https://gesund.bund.de/en/icd-code-search/t05-1)

T05.2 [Traumatic amputation of both arms [any level]](https://gesund.bund.de/en/icd-code-search/t05-2)

T05.3 [Traumatic amputation of both feet [any level]](https://gesund.bund.de/en/icd-code-search/t05-2)

T05.4 [Traumatic amputation of one foot and other leg [any level. except foot]](https://gesund.bund.de/en/icd-code-search/t05-1)

T05.9 [Multiple](https://gesund.bund.de/en/icd-code-search/t05-1) traumatic amputations. unspecified

**Supplemental table 2 Surgical procedures**

**Nerve compression disorders**

ACC43 Transposition of peripheral nerve – ulnar nerve

ACC51 Decompression av peripheral nerve – median nerve

ACC52 Decompression av peripheral nerve – radial nerve

ACC53 Decompression av peripheral nerve - ulnar nerve

ACC54 Decompression av peripheral nerve – peroneal nerve

ACC55 Decompression av peripheral nerve – tibial nerve

ACC56 Decompression av peripheral nerve – sciatic nerve

ACC59 Decompression av peripheral nerve – other or unspecified nerve

**Nerve injuries and neuroma**

ACA12 Exploration of peripheral nerve – radial nerve

ACA13 Exploration of peripheral nerve – ulnar nerve

ACA14 Exploration of peripheral nerve – peroneal nerve

ACA15 Exploration of peripheral nerve – tibial nerve

ACA16 Exploration of peripheral nerve – sciatic nerve

ACA19 Exploration of peripheral nerve – other or unspecified nerve

ACB21 Suture of peripheral nerve – median nerve

ACB22 Suture of peripheral nerve – radial nerve

ACB23 Suture of peripheral nerve – ulnar nerve

ACB24 Suture of peripheral nerve – peroneal nerve

ACB25 Suture of peripheral nerve – tibial nerve

ACB26 Suture of peripheral nerve – sciatic nerve

ACB29 Suture of peripheral nerve – other or unspecified nerve

ACC12 Transection of peripheral nerve – radial nerve

ACC19 Transection of peripheral nerve - other or unspecified nerve

ACC21 Reconstruction av peripheral nerve – median nerve

ACC22 Reconstruction av peripheral nerve – radial nerve

ACC23 Reconstruction av peripheral nerve – ulnar nerve

ACC24 Reconstruction av peripheral nerve – peroneal nerve

ACC25 Reconstruction av peripheral nerve – tibial nerve

ACC26 Reconstruction av peripheral nerve – sciatic nerve

ACC29 Reconstruction av peripheral nerve - other or unspecified nerve

ACC42 Transposition of peripheral nerve – radial nerve

ACC49 Transposition of peripheral nerve - other or unspecified nerve

ZZK00 Nerve graft
